# Two hours in Hollywood: A manually annotated ground truth data set of eye movements during movie clip watching

**DOI:** 10.16910/jemr.13.4.5

**Published:** 2020-07-27

**Authors:** Ioannis Agtzidis, Mikhail Startsev, Michael Dorr

**Affiliations:** Technical University of Munich, Germany

**Keywords:** Eye tracking, eye movement, gaze, smooth pursuit, eye movement classification, hand-labelling, movie viewing

## Abstract

In this short article we present our manual annotation of the eye movement events in a subset of the large-scale eye tracking data set Hollywood2. Our labels include fixations, saccades, and smooth pursuits, as well as a noise event type (the latter representing either blinks, loss of tracking, or physically implausible signals). In order to achieve more consistent annotations, the gaze samples were labelled by a novice rater based on rudimentary algorithmic suggestions, and subsequently corrected by an expert rater. Overall, we annotated eye movement events in the recordings corresponding to 50 randomly selected test set clips and 6 training set clips from Hollywood2, which were viewed by 16 observers and amount to a total of approximately 130 minutes of gaze data. In these labels, 62.4% of the samples were attributed to fixations, 9.1% – to saccades, and, notably, 24.2% – to pursuit (the remainder marked as noise). After evaluation of 15 published eye movement classification algorithms on our newly collected annotated data set, we found that the most recent algorithms perform very well on average, and even reach human-level labelling quality for fixations and saccades, but all have a much larger room for improvement when it comes to smooth pursuit classification. The data set is made available at https://gin.g-node.org/ioannis.agtzidis/hollywood2_em.

## Introduction

In recent years eye tracking has gained further popularity in various fields, and has been applied in increasingly unconstrained scenarios, both in research and commercial applications. These new fields of application move away from stimuli that use clearly defined targets on a monitor and towards more naturalistic content and environments (e.g. movies, virtual reality, everyday life).

In these more naturalistic set-ups, eye movement classification algorithms that were developed with static stimuli in mind, mostly relying on simple statistics such as speed [1] or dispersion [2], are not sufficient anymore, as they fail to account for the more complex and dynamic eye movement patterns. Owing to this, several more elaborate algorithms have been developed [3, 4, 5, 6] in order to overcome the weaknesses of the earlier approaches when applied to dynamic contexts.

For any algorithm, however simple or complex, the question of evaluating its performance is no less vital. Such evaluation is typically performed against some form of “ground truth”. In the case of experiments with dynamic natural stimuli, the decision about which eye movement type should be assigned becomes more difficult, as the distinction between classes is not always clear-cut. For example, in dynamic scenes (e.g. movies), unlike during static scene viewing (e.g. photographs), viewers tend to make smooth pursuit eye movements, and many of the commonly used eye movement classification algorithms do not distinguish fixations and saccades from smooth pursuit. Therefore, in these set-ups, and especially for potentially ambiguous cases, the gold standard is considered to be manual annotation [7, 8, 5]. However, manual labelling is a tedious and time-consuming process, which can require between 10 s to one minute of labour for 1 s of gaze recording, depending on the stimulus domain [9, 10]. Therefore, manually annotated data sets tend to be limited in size, typically varying from a couple of minutes to ca. half an hour [11, 12, 7, 8, 10]. To the best of our knowledge, only one published data set of manually annotated eye movements spans several hours [9].

However, in order to better train and optimise parameter-rich algorithms, a collection of large and diverse data sets is vital. The data set of [9], for instance, contains gaze data recorded during free viewing of dynamic natural scenes (e.g. a duck flying across a river), and is not on its own sufficient to cover all possible (or even frequently occurring) viewing scenarios.

To help overcome this problem and provide a more diverse set of viewing conditions, we here present a large-scale manual annotation of eye movements – fixations, saccades, and pursuits – in a data set of eye tracking recordings during Hollywood movie clip viewing. The movie clips were displayed on a computer monitor and the gaze was recorded with a tower-mounted eye tracker system that employed a chin rest (to eliminate head movement) and reported gaze locations in the coordinate system of the monitor. The recordings for a total of 56 clips are included in our data set, split into a large test set (50 clips) and a smaller training set (6 clips): The latter is not intended for full-scale model training, but could rather serve for final classification algorithm parameter tuning. Such a pipeline would ensure that the algorithms get a fair chance to be adapted to the recordings similar to the test set (same stimuli domain and recording equipment), but still independent of it. The stimuli clips (with their corresponding recordings) were selected from the larger Hollywood2 eye tracking data set [13], and each subset was randomly drawn from the respective test and training portions of the original data set. In total, the annotated gaze data span 130 minutes.

In our data set, apart from the more common fixations and saccades, we also labelled smooth pursuit (SP) eye movements. SP is an important eye movement for the comprehension of motion since it keeps targets that move relative to the observer foveated. Also research evidence indicates that different functional areas of the brain subserve SP [14, 15, 16] in comparison to fixations and saccades [17, 18, 19]. Pursuing moving targets is vital for the comprehension of Hollywood movie scenes due to the extensive camera and object motion present in them. This importance is reflected in the high percentage of SP (almost a quarter of the viewing time) in our labelled data. However, annotating SP is not always straightforward, and the addition of this eye movement type makes manual labelling more challenging.

In the absence of objective and universally accepted ground truth [20], the quality of eye movement labellings is mainly determined by their internal consistency. We therefore provided clear eye movement definitions (presented in the next section) and each gaze sample was processed consecutively by two individual annotators. On the first pass, an annotator went through the laborious and time-consuming process of labelling all the gaze samples based on rudimentary and incomplete algorithmic suggestions. Despite best efforts, any manual process of the scale presented here likely introduces occasional errors. Therefore, the labelling was reviewed independently by an expert annotator (first author) who was presented with the previously annotated data and was free to make changes wherever he felt the eye movement definitions were violated. Based on the annotated data set, we present several basic eye movement statistics along with the evaluation of the performance of 15 classification algorithms from the literature.

## Methods

**Figure 1.**
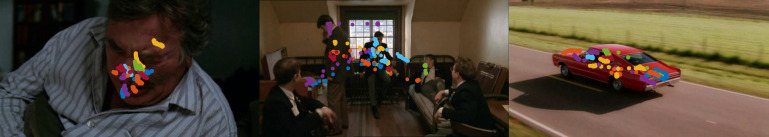
Sample scenes from the Hollywood2 data set overlaid with 600 ms of gaze samples. The gaze pattern of each participant is visualised with a unique colour. The left and right snapshots show scenes with substantial motion and they contain 72% and 59% of smooth pursuit respectively. The high prevalence of pursuit is also visible by the elongated coloured lines (representing consecutive gaze samples) that are oriented along the direction of the motion. The middle snapshot does not contain smooth pursuit and the trace directions are more varied.

Before explaining the labelling process in more detail, we will briefly present the unlabelled data set, upon which we built our current work. The Hollywood2 data set [13] was recorded, as its name suggests, with Hollywood movies (movie excerpts, to be precise) as stimuli and it contains ca. 70 hours of gaze recordings. Some example scenes overlaid with gaze samples of the different observers are provided in Figure 1. The purpose of the data set was action recognition through eye movements, and the pool of 16 eye tracking experiment participants was split into two groups. The task of the “active” subgroup (12 subjects) was to assign one of the 12 action classes to each video clip. The “free viewing” subgroup (4 subjects) had no task and was simply watching the video clips. The participants’ head was stabilised with a chin rest and the eye movements were recorded monocularly from the dominant eye at 500 Hz with an SMI iView X HiSpeed 1250 eye tracker. A relatively high eye tracking accuracy of 0.75 degrees was achieved via a 13-point calibration procedure at the beginning of each recording block, plus a validation step at the end – if validation accuracy fell outside these limits, the data were discarded.

### Eye movement definitions

In order to avoid potential confusion about the meaning behind each labelled class of eye movements, we provide the definitions that were used during our manual annotation. These are similar to those used in [9]. The only difference from the definitions used in that work is contained in our smooth pursuit definition, which now explicitly accounts for video object motion on the monitor that is caused by camera motion – something that almost never occurred in the [9] data.

Fixation: A period of time where the gaze is relatively stationary on the monitor (and thus relative to the observer) as reported by the eye tracker and does not follow a moving object.

Saccade: A jump to a different on-screen position without any specific amplitude or speed threshold being imposed. The end of a saccade was marked when the gaze had stabilised again. Because of the difficulty in defining post-saccadic oscillations (PSOs) and because of their diverse shapes and durations [21], PSOs were considered parts of the corresponding saccades in our annotations.

Smooth pursuit: A period of time where the gaze was smoothly moving and was following an on-screen moving object (either due to its own movement or camera motion) with roughly matching velocity (speed and direction) in screen coordinates. If the gaze was moving smoothly but without a potentially corresponding object motion, this part of the recording was labelled as a fixation, with the assumption that it was either drifting or affected by some recording artefacts (e.g. reported gaze drifts due to pupil diameter changes [22]).

Noise: Parts of the gaze signal that do not fulfil any of previous eye movement definitions (hence could also be interpreted as the “other” label or similar). These intervals include blinks (together with the often-occurring up- and downwards saccade-like patterns around them), parts of the gaze recordings that fall outside the monitor, intervals where the eye tracker reported zero confidence, and physically implausible eye movements. For the purpose of this manuscript, blinks were labelled as noise (and not separately coded) because they are not always distinguishable from tracking loss in the absence of the camera signal of the videooculographic tracker. Despite the inability to perfectly judge whether a blink took place based on the point-of-regard signal alone, it is common practice in the eye tracking community to extract blinks based on the related signal artefacts typically observed in video-oculography (large downwards and upwards saccade-like patterns surrounding periods of lost tracking), and performing such analysis should be relatively straightforward based on the noise labels we provide.

### Labelling procedure

**Figure 2.**
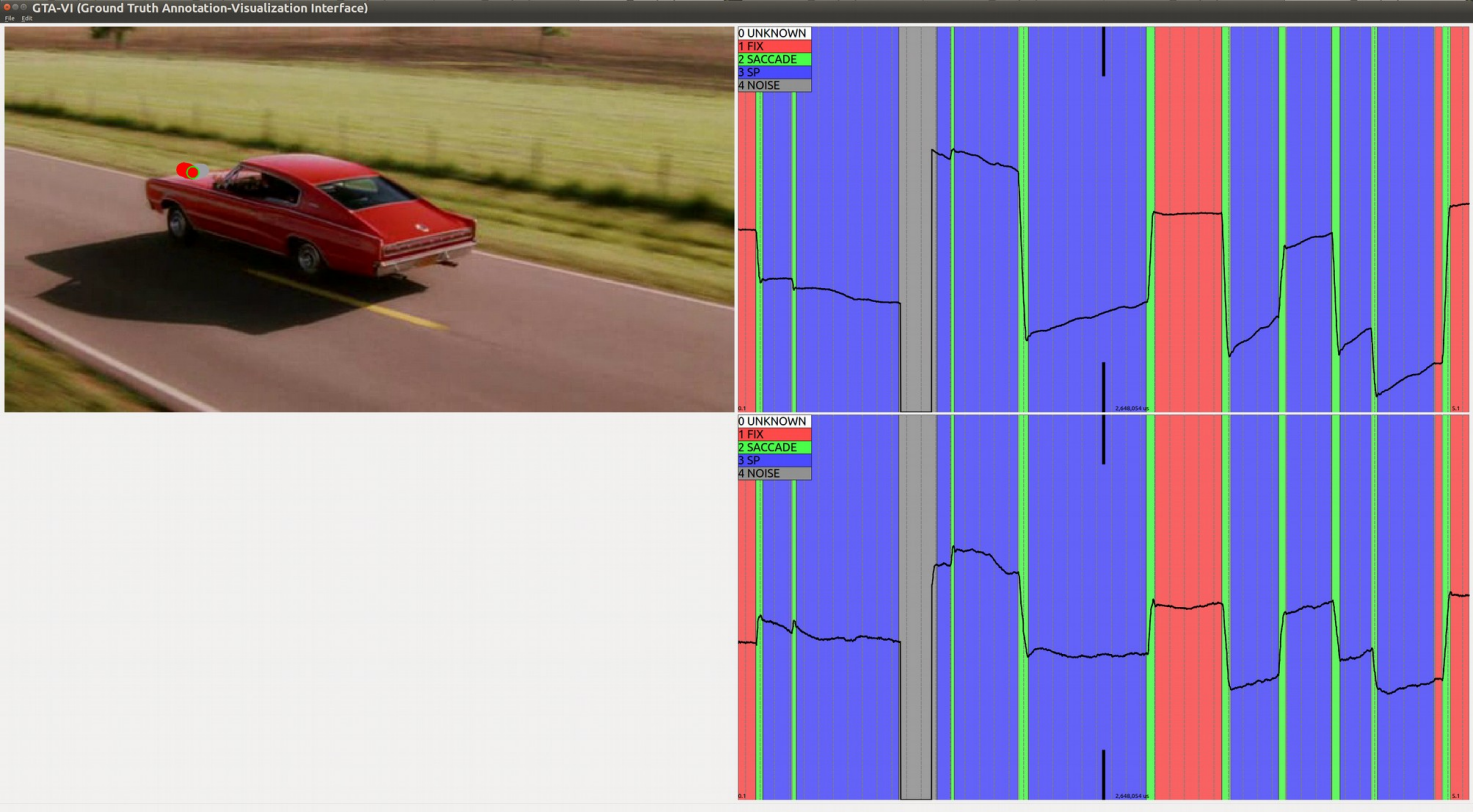
Example screenshot of the tool as it was used during labelling. The two panels on the right are used during the labelling procedure in order to change the borders of the colour-coded areas depending on the x and y coordinates (black lines). The still frame here (top-left panel) comes from the same video as the rightmost frame in Figure 1. For the specific participant here, most of the samples are labelled as SP (blue boxes) due to the gaze following either camera or object motion.

For manual annotation we used the software developed in [23], which presents the video clip together with the participant’s gaze in four panels. An example screenshot of the tool as it was used during labelling is presented in Figure 2. The main panel displays the video stream overlaid with 200 ms of gaze (i.e. samples within 100 ms from the “current” one). The two panels to its right display the x and y coordinates of the gaze signal along with colour-coded boxes that represent different eye movement classes. These boxes can be adjusted, added, or deleted by the human annotators, and are, therefore, the main interaction point with the interface. The last panel (located below the video panel) is optional and was not used in this experiment. Both the labelling tool and the hand-labelled data set in this work use the text based ARFF files; more details about the file format can be found in [23, 9].

For the labelling of the eye movements, two human annotators worked on each gaze recording one after the other. The first labeller was a paid student at the Technical University of Munich, working part-time (8 h/week for 22 weeks), who obtained basic knowledge about eye movements from following a relevant course, as well as additional clarifications from the authors. This first annotator was also provided with representative examples for the eye movement definitions from Section “Eye movement definitions” in action in the context of the labelling interface. During the full duration of the labelling process, experts were available to answer any questions. Randomly chosen annotated files were periodically visually inspected by the authors, and feedback was provided to the annotator.

To speed up the labelling process, the gaze files were pre-segmented with the I-VVT algorithm [24] with default parameters before being presented to the first annotator. By providing the automatically labelled intervals, even if those were poorly aligned with actual eye movements, the task of the annotator was simplified to mainly merging intervals and correcting their temporal locations, instead of constantly adding new intervals one by one and then correcting their borders. Such pre-annotation has been shown to provide considerable manual labelling speed-up, though researchers have to take extra care in order to avoid biasing the results [9]. Due to using the I-VVT algorithm instead of a more elaborate approach (see Section “Evaluation of classification algorithms”), the labeller could not leave its labels uncorrected: The outputs of I-VVT on our data were very noisy (see Table 1 for final agreement), meaning that the first annotator had to carefully inspect the full file. Any potential bias introduced by the algorithmic pre-segmentation therefore would have been small.

The second annotator (the first author) then performed the final pass over all the gaze files. The second labeller could freely modify the gaze event intervals wherever it was deemed necessary. We consider the labels yielded by this annotator as final, though the work of both annotators is included in our data for transparency.

## Results

### Basic statistics

The hand-labelling process for our data set required approximately 230 hours of labour, which were roughly split into 170 hours for the novice labeller and 60 hours for the expert. Overall, the labelled data set contains 14,643 fixations, 15,082 saccades, and 5649 SP episodes, with the eye movement types representing 62.4%, 9.1%, and 24.2% of the total gaze samples, respectively (the rest were marked as noise).

**Figure 3.**
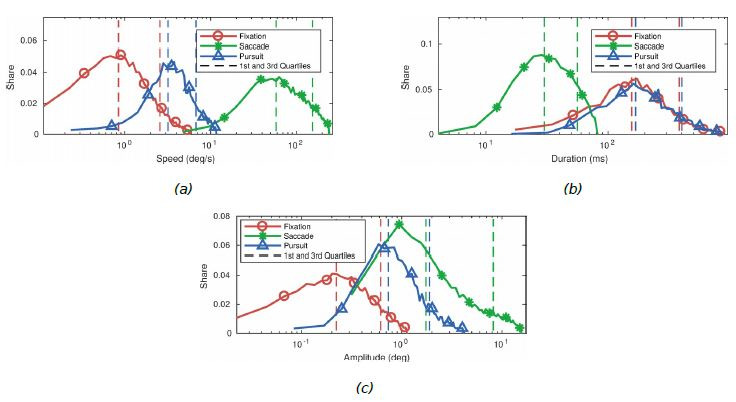
Distributions of speed (3a), duration (3b), and amplitude (3c) for the three labelled eye movement classes. Note the logarithmic scale of the x axis (chosen due to the large range of the reported statistics for the three classes). To facilitate the comparison between the distributions, we visualise the first and third quartiles of each distribution as vertical dashed lines.

To better understand the characteristics of the three labelled eye movement types in this data set, we present in Figure 3 the distributions of their speeds, durations, and amplitudes. The amplitude was computed as the distance between the first and the last samples in each eye movement interval, while the speed was computed by dividing the amplitude by the respective interval duration. Note that the horizontal axes of the plots are in logarithmic units; this non-linearity makes direct comparison more difficult, but allows for a better visualisation of the large value range that is spanned by the distributions of the presented attributes for the defined eye movements classes.

From Figure 3a we can see that saccades, being much faster than the two other classes, are clearly distinguishable by considering speed alone. Smooth pursuits, on the other hand, are expectedly faster than fixations on average, but there is a substantial overlap between the two classes in terms of their average speed, as it is evident from the quartile lines (vertical dashed lines) in the figure. This overlap makes the distinction between drifting fixations and SP more challenging, at least when purely speed-based thresholding is attempted.

Examining event durations (Figure 3b), saccades are again clearly separated from the other two types as their maximum duration does not exceed 100 ms in our data. By contrast, 75% of fixation and SP intervals lasted longer than 160 ms, and their overall duration distributions almost perfectly overlap.

Finally, the amplitude distributions of the three eye movement types (related to the dispersion feature used by classifiers) are presented in Figure 3c. Here, a fair separation between fixations and saccades would be possible with a single threshold in the absence of SP. The distribution related to the latter class, however, significantly overlaps with the amplitude distributions of both fixations and saccades, thus making a good separation among the three impossible with simple thresholding.

### Evaluation of classification algorithms

Data sets such as this one, apart from providing valuable insights into the eye movement characteristics, also serve as an essential tool for the development of algorithms that automatically segment the gaze signal into eye movements. The annotated data can be used as basis for the validation and optimisation of rule-based algorithms, but also as a training set for the machine learning and deep learning approaches, which have offered significant performance increases in many fields in recent years.

Here, we present the evaluation results for 15 publicly available algorithms (one of the entries is a post-processing result of another). We do not provide the details for the tested algorithms, as the data set itself is the main focus of this article, but we refer the reader to the original papers cited next to each entry in Table 1. In brief, we tested a variety of algorithms, ranging from simple thresholding techniques to deep learning approaches. Most of the evaluated algorithms label smooth pursuit as well as fixations and saccades. For the evaluation we used the F1 score as quality metric, which is the harmonic mean of precision and recall. The F1 score was used for the evaluation of both sample- and event-level (i.e. continuous sequences of samples with the same label) matching between the hand-labelled ground truth and the algorithm outputs. For event-level evaluation, we employed the algorithm of [25] for event matching, where the ground-truth events are matched with the earliest intersecting algorithmically labelled event and only one-to-one association is allowed.

**Table 1.** Evaluation results for 15 publicly available eye movement classification algorithms. The order of presentation is based on the average F1-score.

		Sample-level F1			Event-level F1		
Model	F1 average	Fixation	Saccade	SP	Fixation	Saccade	SP
1D CNN-BLSTM [6]	**0.787**	**0.872**	**0.827**	**0.680**	0.808	**0.946**	0.588
sp tool + [26]	0.755	0.853	0.816	0.617	0.820	0.905	0.516
REMoDNaV [4]	0.748	0.779	0.755	0.622	0.784	0.931	**0.615**
sp_tool [27]	0.703	0.819	0.815	0.616	0.587	0.900	0.483
[28]	0.685	0.832	0.796	0.373	0.821	0.884	0.403
[3]	0.647	0.796	0.803	0.317	0.807	0.886	0.274
[29]	0.601	0.824	0.729	0.137	**0.845**	0.826	0.243
I-VMP [30]	0.564	0.726	0.688	0.564	0.503	0.563	0.338
I-KF [31]	0.523	0.816	0.770	–	0.748	0.803	–
I-VDT [24]	0.504	0.813	0.700	0.136	0.557	0.559	0.263
I-HMM [32]	0.480	0.811	0.720	–	0.646	0.700	–
I-DT [2]	0.473	0.803	0.486	–	0.744	0.802	–
I-VT [2]	0.432	0.810	0.705	–	0.520	0.555	–
I-VVT [24]	0.390	0.751	0.705	0.247	0.061	0.555	0.023
I-MST [33]	0.385	0.793	0.349	–	0.590	0.576	–

Cells marked with “–” denote an eye movement type that was not classified by the given algorithm and therefore no evaluation was possible.

All the algorithms in Table 1 were evaluated as provided by the authors, with their default parameters, on the test set part (50 video clips) of our hand-labelled data set. The implementation of the algorithms starting with “I-” was provided by the toolbox of [34]. The authors of [3] did not make the source code of their algorithm publicly available, so our re-implementation^1^ of this algorithm was used.

The earliest algorithms in this table (namely I-KF, I-HMM, I-DT, I-VT, and I-MST) were designed with the assumption that the experimental stimuli are exclusively static and, therefore, they do not label SP (the eye movement accounting for a quarter of the samples in our data set). As a result, these algorithms achieved some of the lowest (average) scores. It is worth mentioning that most of eye movement filters that are provided by the eye tracker manufacturers rely on these algorithms or their variations, and are made available as-is through closed-source distributions. Our evaluation results, therefore, indicate that the outputs of such systems cannot be always trusted to deliver adequate labels, in particular when dynamic stimuli are utilised.

All the more recent algorithms that are evaluated here have the ability to classify SP. However, the approaches that only rely on simple rules (namely I-VVT, I-VDT) yielded very low scores, likely due to significant overlap between the basic statistics of the different eye movements as demonstrated in the previous section, cf. Figure 3. Here, it should be noted that the I-VVT algorithm was used to pre-annotate the data set in order to speed-up the labelling process. From the results table, it becomes evident that the end result of the hand-labelling process is not comparable to the I-VVT suggestions, as this algorithm only ranked second to last.

Our clustering-based SP classification approach [27] achieved high sample-level F1 scores, but its known weakness is the erroneous fragmentation of long events in the ground truth into shorter ones [9]. For this reason, we applied our recent hidden Markov model (HMM) based label smoothing technique [26] to its outputs. The smoothing model was trained on the outputs of this algorithm for the training subset of the data (6 clips). It was then used to improve the labels of the same algorithm [27] on the outputs on the 50-clip test part. After the smoothing operation, the average F1-score of the algorithm increased 5%, while fixation event-level F1 shot up by 23%.

The newest algorithms we tested [6, 4] achieved the highest average F1-scores, indicating their capabilities for robust automatic analysis of unseen large-scale data corpora. Nevertheless, their performance in terms of SP classification was significantly lower than that for fixations or saccades, demonstrating the difficulty of classifying this eye movement type and pointing out the necessity for further improvements in this domain. In fact, all of the evaluated algorithms demonstrated lower SP classification performance when compared to either fixations or saccades.

## Discussion

### Data set statistics

As we have presented in the previous section, a large part of the viewing behaviour is devoted to SP: This eye movement accounts for almost a quarter of the viewing time, on average across stimuli and observers. This is a much higher figure than the previously reported 11% and 9.8% for the video free-viewing GazeCom [9] and 360-degree video [10] data sets, respectively. The figure is, however, almost two times lower than the 52.2% for the video viewing part of the data set from [7], where participants were explicitly instructed to follow moving objects with their eyes. Also, while the overall viewing time in this data set is much lower than in GazeCom (ca. 2.2 h vs. 4.7 h), the amount of recorded smooth pursuit is almost identical between the two (ca. 32 min vs. 31 min) because of the much higher proportion of pursuit in our data set.

Based on the negligible difference in the SP share between the “active” and the “free viewing” groups in the current data set (24% vs. 24.3%), we conclude that the differences in the SP amount between the current data set and GazeCom or 360-degree data set in [10] likely originates from the different stimuli types (Hollywood movie clips vs. naturalistic videos), and not from the task performed by the observers (free-viewing vs. action recognition).

**Figure 4.**
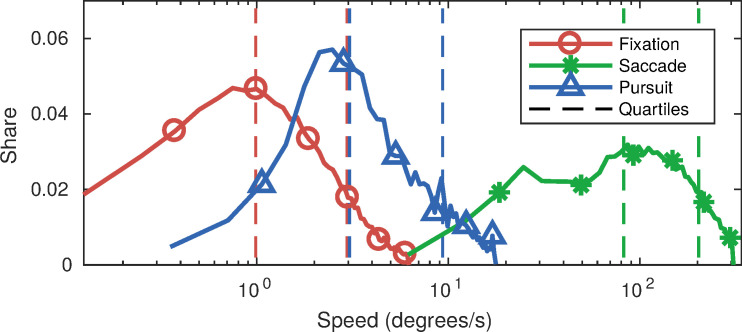
Event speed distribution for the GazeCom data set. Note the logarithmic scale of the x axis (chosen due to the large range of the reported speed for the three classes). The figure presented here is a reproduction of Figure 4 from [9].

Comparing the gaze event speed distributions in our data set (Figure 3a) and the equivalent statistic in the GazeCom data set (depicted in Figure 4) one can observe that they are very similar in shape. Fixations and saccades are easily separable from one another, with SP speeds somewhere in-between the two other eye movement types, overlapping with both.

Examining the distributions more closely, however, reveals that the eye movement speeds are lower in Hollywood2 (in comparison to GazeCom) across the board. For fixations, the difference is very small and could be explained by the different eye tracking systems used for their recording (first and third quartiles differ by less than 0.5 deg/s between the two data sets). For SP, the difference is also relatively small (5.4 vs 7.0 deg/s for the first quartile and 6.8 vs 9.3 deg/s – for the third, comparing the Hollywood2 subset vs. the GazeCom, respectively). This effect likely arises from the different properties of the moving targets in the two data sets, as the SP speed typically closely follows the speed of the pursued target [35]. Finally, for saccades we see a substantial difference in gaze speed between the two data sets: The first quartile reaches 52 deg/s for the current data set vs. 82 deg/s for the GazeCom (note that these are overall and not peak speeds); the third quartiles reach 154 deg/s vs. 202 deg/s, respectively. These differences can be potentially explained by the more centre biased gaze patterns in Hollywood2 than in GazeCom [28], which would result in lower saccadic amplitudes and, therefore, lower saccadic speeds [36], despite the similar monitor sizes in the two experiments.

### Combination with other data sets

Though the data set that we presented here does not attempt to cover all the conditions that humans experience in their everyday lives, it can be combined with other published data sets in order to achieve a more comprehensive superset, thus allowing to examine human eye movements in a more diverse set of paradigms, possibly in combination with the corresponding visual attention allocation mechanisms. Studying the latter via the means of computer vision (e.g. saliency prediction) requires large amount of diverse data in a variety of contexts, to which this work is contributing as well. In terms of diversity, the data set of [7], for example, despite its small overall duration, contains three stimulus categories that span moving dots, still images, and videos.

For a larger-scale analysis, e.g. the GazeCom [9] data set and the data set from [10] can supplement the data presented here, resulting in many hours of manually annotated data of human behaviour in dynamic scenes, either natural or cinematic, presented on a monitor or a head-mounted display that allows free head motion.

Large and diverse saliency data sets [37, 38, 39] can further help us understand the allocation of attention, but the data that is typically published is somewhat limiting, as they only provide saliency maps or scanpaths at best (i.e. not the raw gaze tracking data, but already processed by some standard algorithm or a filter built into the eye tracker [37, 40, 41]). Only few exceptions can be named, among them – the eye-1 data set by [42] and the fully processed Hollywood2 data set in [43], where several eye movement classes (including fixations and smooth pursuit) were algorithmically labelled.

Also the combination of various human eye movement data sets that represent diverse viewing scenarios can help us better understand the human viewing behaviour and develop improved algorithms. These, in turn, enable a higher quality automatic analysis of fMRI [44, 45] and clinical data [46, 47], which could offer a better understanding of the neural mechanisms that drive human vision. These large scale analyses would have been impossible if we had to rely on manual labour only. Finally, more intuitive and comfortable gaze based interfaces [48, 49] can be designed based on these more diverse experimental data, e.g. by deriving and using the properties of the naturally occurring eye movements in various scenarios.

## Conclusion

In this article we presented a large-scale hand-labelled ground truth data set of eye movements that used Hollywood movie clips as stimuli. Based on these labels, we then presented some basic eye movement characteristics not only for fixations, saccades, but also smooth pursuits. Afterwards, we evaluated the classification performance of 15 eye movement labelling algorithms that varied from classical to state-of-the-art. The data set and results presented here contribute towards a better understanding of visual behaviour patterns in naturalistic contexts.

### Ethics and Conflict of Interest

The authors declare that the contents of the article are in agreement with the ethics described in http://biblio.unibe.ch/portale/eli-brary/BOP/jemr/ethics.html and that there is no conflict of interest regarding the publication of this paper.

### Acknowledgements

This research was supported by the Elite Network Bavaria, funded by the Bavarian State Ministry for Research and Education.

## Footnotes

http://michaeldorr.de/smoothpursuit/larsson_reimplementation.zip
